# Power of the Plug Hat

**Published:** 1885-12

**Authors:** 


### POWER OF THE PLUG HAT.

The plug hat is virtually a sort of social guarantee for the preservation of peace and order. He who puts one on has given a hostage to the community for his good behavior. The wearer of a plug hat must move with a certain sedateness and propriety. He cannot run, or jump, or romp, or get into a fight except at the peril of his head-gear. All the influences of the beaver tend toward respectability. He who wears one is obliged to keep the rest of his body in decent trim, that there may be no incongruity between head and body. He is apt to become thoughtful through the necessity of watching the sky whenever he goes out. The chances are that he will buy an umbrella, which is another guarantee for good behavior, and the care of hat and umbrella—perpetual and exacting as it must ever be—adds to the sweetness of his character. The man who wears a plug hat naturally takes to the society of women, with all its elevated tendencies. He cannot go hunting or fishing without abandoning his beloved hat, but in the moderate enjoyment of croquet or lawn-tennis he may sport his beaver with impunity. In other words, the constant use of a plug hat makes a man composed in manner, quiet and gentlemanly in conduct, and a companion of ladies. The inevitable result is prosperity, marriage and church membership.

Coal dealers and grocers never turn highway robbers. They know how to accomplish the same thing safer on the low weigh.

